# Clinical and Electrophysiological Predictors of Isthmus Dependency in Atrial Flutter

**DOI:** 10.3390/diagnostics15091095

**Published:** 2025-04-25

**Authors:** Lyuboslav Katov, Sonja Reiländer, Alyssa Schlarb, Federica Diofano, Deniz Aktolga, Yannick Teumer, Carlo Bothner, Wolfgang Rottbauer, Karolina Weinmann-Emhardt

**Affiliations:** Department of Cardiology, Ulm University Heart Center, Albert-Einstein-Allee 23, 89081 Ulm, Germany; lyuboslav.katov@uniklinik-ulm.de (L.K.); sonja.reilaender@uni-ulm.de (S.R.); alyssa.krafft@uni-ulm.de (A.S.); federica.diofano@uniklinik-ulm.de (F.D.); deniz.aktolga@gmail.com (D.A.); yannick.teumer@uniklinik-ulm.de (Y.T.); carlo.bothner@uniklinik-ulm.de (C.B.); wolfgang.rottbauer@uniklinik-ulm.de (W.R.)

**Keywords:** cavotricuspid isthmus-dependent atrial flutter, typical atrial flutter, atypical atrial flutter, type I ECG, type II ECG, predictors

## Abstract

**Background**: Atrial flutter (AFL) is a macro-reentrant tachycardia classified as cavotricuspid isthmus (CTI)-dependent or non-CTI-dependent based on its reliance on the CTI for conduction. CTI dependence can present as type I ECG (sawtooth flutter waves in inferior leads and positive P-waves in V1) or type II ECG (absence of these characteristics). This study aimed to identify clinical and electrophysiological parameters to improve CTI dependence prediction in AFL. **Methods**: Patients at the Ulm University Heart Center between 2010 and 2019 with AFL undergoing electrophysiological studies and ablation were enrolled. Clinical and electrophysiological parameters such as age, gender, prior comorbidities, interventions, and medication use were analyzed. **Results**: The study included 383 patients, with 70% presenting with type I ECG AFL. CTI dependence was observed in 242 (90.3%) type I ECG patients and 52 (45.2%) type II ECG patients. CTI-dependent AFL patients were younger and had fewer comorbidities. Predictors for CTI dependence in type I ECG included male gender (*p* = 0.006), absence of beta-blocker use (*p* = 0.031), no prior atrial fibrillation (*p* = 0.035), and no prior pulmonary vein isolation (*p* < 0.001). In type II ECG, predictors for CTI dependence included younger age (*p* = 0.016), male gender (*p* = 0.007), absence of arterial hypertension (*p* = 0.036), and longer atrial cycle length (*p* < 0.001). **Conclusions**: Identifying clinical and electrophysiological parameters enhances the ability to predict CTI dependence in AFL, offering valuable insights for tailored diagnostic and therapeutic approaches. Coupling these parameters with ECG findings holds promise for refining prediction accuracy and optimizing patient care.

## 1. Introduction

Atrial flutter (AFL) is a common supraventricular arrhythmia characterized by a macro-reentrant circuit in the atria, with an incidence of 88 per 100,000 person-years [1]. According to its pathophysiologic mechanism, AFL can be classified into cavotricuspid isthmus (CTI)-dependent AFL, commonly referred to as typical AFL, and non-CTI-dependent AFL, known as atypical AFL [2]. Among these, typical AFL is the most prevalent and clinically relevant form. This arrhythmia is defined by a reentrant circuit that depends on the CTI, an anatomical region in the right atrium bordered by the tricuspid valve and the inferior vena cava (IVC). The CTI plays a pivotal role in the pathophysiology of typical AFL due to its slower conduction velocity compared to adjacent atrial tissue, which facilitates the formation and maintenance of a stable reentrant circuit with a cycle length (CL) between 200 and 260 ms [3,4,5,6,7]. Typical, counterclockwise (CCW) AFL is identified on electrocardiography (ECG) by its characteristic pattern of negative sawtooth flutter waves in the inferior leads (II, III, and aVF) and positive flutter waves in lead V1, referred to as the type I ECG pattern. In contrast, atypical AFL is not CTI-dependent and presents as a heterogeneous group of macro-reentrant arrhythmias, characterized more commonly by their inability to meet the criteria for a type I ECG and instead exhibiting a type II ECG pattern [3,8]. The reentrant circuit often involves other anatomical structures other than the CTI, such as atrial scars caused by prior surgeries, catheter ablations, or degenerative processes. Atypical AFL generally presents with less distinct ECG features, making its diagnosis more complex [3,6,9].

Clinically, AFL is associated with a variety of symptoms, including palpitations, dyspnea, fatigue, and dizziness, which can significantly impair a patient’s quality of life. AFL also carries a risk of serious complications, including new onset of atrial fibrillation (AF) (50%), stroke, thromboembolism, and tachycardia-induced cardiomyopathy [10,11,12,13,14]. While the diagnosis of AFL often begins with a 12-lead ECG, definitive confirmation of the arrhythmia mechanism and CTI dependency typically requires invasive electrophysiological studies (EPSs) [3]. As CTI dependence can be reliably confirmed through EPSs, non-CTI-dependent mechanisms remain more diverse, often involving macro-reentrant circuits around left atrial scars (post-ablation or surgical), the mitral isthmus, or septal regions, and require advanced mapping techniques for precise characterization [10,15].

Catheter ablation, specifically CTI ablation for typical AFL, is the preferred treatment due to its high success rates and a recurrence risk of less than 10% [16,17,18]. In patients with atypical, non-CTI-dependent AFL, the variability in circuit location and mechanism increases procedural complexity and reduces success rates, necessitating advanced mapping techniques and individualized approaches for accurate identification and effective treatment [16]. However, identifying predictors of CTI dependency in AFL is critical to improving diagnostic efficiency, guiding treatment decisions, and optimizing patient outcomes. This study aims to analyze clinical, electrophysiological, and ECG parameters associated with CTI-dependent AFL. By identifying reliable clinical predictors, this research seeks to enhance diagnostic accuracy, streamline invasive procedures, and improve the overall management of patients with AFL.

## 2. Materials and Methods

### 2.1. Study Population

**Figure 1:**
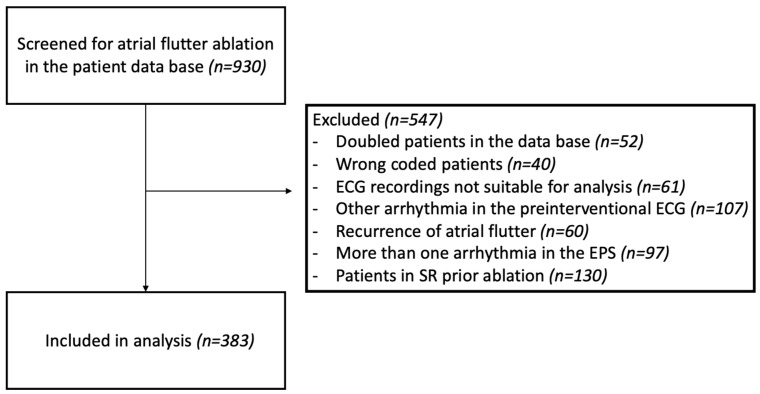
Consort diagram illustrating patient selection. Of 930 patients screened for AFL ablation, 547 were excluded due to duplicates, incorrect coding, unsuitable ECG recordings, other arrhythmias on pre-interventional ECG, recurrence of atrial flutter, multiple arrhythmias during EPSs, and sinus rhythm prior to ablation. A total of 383 patients were included in the final analysis. AFL, atrial flutter; EPS, electrophysiological study.

For this retrospective cohort study, we screened 930 consecutive patients who underwent an EPS at Ulm University heart center between January 2010 and December 2019, including cases encoded as typical (ICD-10: I48.3) or atypical atrial flutter (ICD-10: I48.4) in the clinical data system. Additional cases were identified through a review of catheterization lab intervention plans. Informed consent was obtained from each patient. In total, 547 patients of the screened population were not eligible for enrolment for the study due to one or more of the following reasons: multiple listing or wrong coding of cases in the clinical data management system, ECG tracings not suitable for analyzing atrial waves, recurrence of AFL, clinical arrhythmia either not inducible or unstable or with changeable patterns or converted in sinus rhythm and a different mechanism of tachyarrhythmia detected at EPS (Figure 1).

A total of 383 patients presented with stable AFL ECG patterns and were eligible for further analysis. All patients underwent EPSs, and CTI-dependent AFL was confirmed by entrainment mapping and termination of AFL under CTI ablation. Patients’ characteristics, medical history, and ECGs were obtained from medical records prior to EPS and ablation. The study protocol adheres to the ethical guidelines outlined in the Declaration of Helsinki and was approved by the local ethics committee (ethics approval Nr. 324/16).

### 2.2. EPS and Ablation

To categorize atrial arrhythmia in CTI-dependent and non-CTI-dependent AFL, all patients underwent EPSs. Patients with suspected CTI-dependent flutter received a conventional EPS to confirm the macro-reentrant tachycardia in the right atrium. Access to the right atrium was obtained via the right femoral vein, and a steerable decapolar diagnostic catheter (Inquiry, Abbott, formerly St. Jude Medical, St. Paul, MN, USA) was placed in the coronary sinus (CS) or positioned at the tricuspid annulus. To determine the post-pacing interval (PPI), entrainment was performed from the two proximal and two distal electrodes of the diagnostic catheter with a CL slightly shorter than the baseline AFL CL. A return CL equal to or within 20 ms of AFL CL at the CS ostium, the lower free wall of the right atrium (low right atrium‚ LRA), and on the CTI and a concealed entrainment at these sites are considered as CTI-dependent or typical AFL [4].

The direction of typical AFL was determined by the activation pattern of the decapolar catheter at the tricuspid annulus and classified as clockwise (CW) or CCW. After confirming CTI-dependent AFL, a deflectable-tip ablation catheter (Blazer II XP™, 8 mm, Boston Scientific, Marlborough, MA, USA; Thermocool^®^ Celcius, Thermocool^®^ Smarttouch SF, Biosense Webster, Irvine, CA, USA; Therapy™ Cool Flex™, St. Jude Medical, St. Paul, MN, USA; AlCath LT Gold FullCircle, Biotronik, Berlin, Germany) was placed on the CTI and radiofrequency energy was applied at 6 o’clock of the tricuspid annulus in left anterior oblique (LAO) 40° view. The ablation line was established between the tricuspid annulus and the IVC orifice. Termination of AFL confirmed ablation within the macro-reentrant circuit. Directly after completion of CTI ablation and after a waiting time of up to 30 min, a bidirectional block was confirmed by determination of the activation pattern and conduction time from the CS ostium to the LRA and vice versa. If EPS detected non-CTI-dependent AFL, atrial arrhythmia was terminated via electrical direct current cardioversion and ablation was rescheduled using an electroanatomical mapping and ablation system.

### 2.3. ECG Analysis

**Figure 2:**
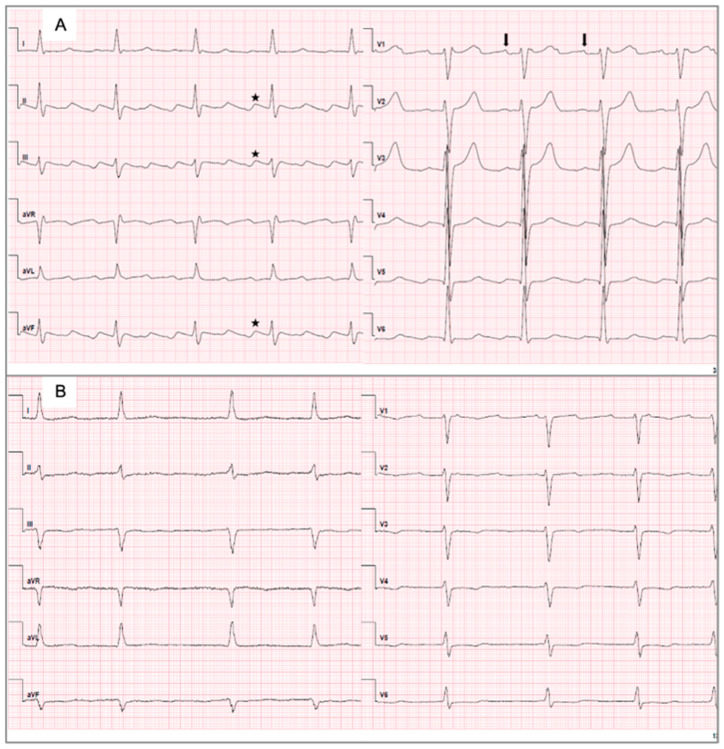
Depiction of a type I ECG, also referred to as typical AFL, and of a type II ECG, also referred to as atypical AFL. (**A**) Type I ECG shows the characteristic sawtooth pattern in II, III, aVF (black stars) and positive P-waves in V1 (black arrows). (**B**) Type II ECG is a flutter ECG that does not fulfill the criteria of a type I ECG. AFL, atrial flutter; ECG, electrocardiogram.

A 12-lead ECG of every patient was recorded prior to the EPS and ablation. All ECGs were independently reviewed by at least two experienced electrophysiologists from a high-volume tertiary electrophysiology center, each with substantial expertise in arrhythmia interpretation. In cases of disagreement, a third independent electrophysiologist was consulted to reach consensus. This rigorous multi-review approach aimed to ensure high diagnostic accuracy and reproducibility in distinguishing between type I and type II ECG patterns. ECGs were considered as type I ECGs (Figure 2A) if the characteristic ECG sawtooth pattern was present in leads II and III, and/or aVF with slow downsloping and a negative segment, followed by a shorter, sharply positive deflection leading to the next downsloping segment, and a positive P-wave in V1. On the contrary, either an inverse sawtooth pattern, with a slow upsloping positive segment followed by a shorter negative deflection, no or no clear sawtooth pattern in the inferior leads, negative or biphasic P-waves or low voltages without clear, identifiable P-waves in V1 were considered as type II ECGs (Figure 2B).

### 2.4. Statistical Analysis

Statistical analyses were performed using SPSS^®^ software (SPSS, V29, Chicago, IL, USA). Categorical variables are described as absolute and relative frequencies, and continuous variables are expressed as mean ± standard deviation (SD). Univariate analyses were performed using a binominal logistic regression model, and predictors that were independently associated with an unusual ECG pattern were analyzed in a multivariate logistic regression model. A *p*-value of <0.05 in the univariate analysis qualified to enter the multivariate model. Groups were compared using a two-sided *t*-test or a Mann–Whitney test, as appropriate. For subgroup analyses, *p*-values reflected overall group differences, and post hoc testing was conducted to identify specific between-group differences. A two-tailed *p*-value of <0.05 was considered statistically significant. Receiver operator characteristic (ROC) analyses were performed to determine the area under the curve (AUC) and the sensitivity/specificity of the predictors. Analysis of variance (ANOVA) tests were conducted to assess differences among multiple groups for continuous variables, while chi-square tests were used for categorical variables to evaluate associations across multiple groups. If ANOVA showed significant differences between groups, additional post hoc tests were performed to determine which specific groups differed from each other.

## 3. Results

### 3.1. Baseline Characteristics

We included 383 patients diagnosed with AFL due to characteristic ECG patterns. The majority of patients were male (300/383 patients, 78.3%), and the mean age of patients was 69.3 ± 11.2 years. On hospital admission, 268 patients (70%) presented a type I ECG and 115 patients (30%) showed a type II ECG. The majority of patients were diagnosed with CTI-dependent atrial flutter (294/383 patients, 77%) (Table 1).

There was a high prevalence of cardiovascular comorbidities, like arterial hypertension (275/383 patients, 72%), diabetes mellitus (89/383 patients, 23%), coronary heart disease (149/383 patients, 39%), and prior AF (145/383 patients, 38%). A total of 185 patients (48.3%) had heart failure (HF), of whom 70 patients (18.3%) were diagnosed with HF with reduced ejection fraction (HFrEF), 61 patients (15.9%) with HF with preserved ejection fraction (HFpEF), and 67 patients (17.5%) with HF with mildly reduced ejection fraction (HFmrEF). The mean left ventricular ejection fraction (LVEF) was 50 ± 15%. A history of prior right or left atrial ablation was present in 3.4% (13/383 patients) of these patients, 9.1% (35/383 patients) of the cohort had prior pulmonary vein isolation, and 16.4% (63/383 patients) of patients had prior heart surgery. Detailed information on patients’ baseline characteristics is provided in Table 2.

#### 3.1.1. Baseline Characteristics of Patients with Type I ECGs and Type II ECGs

In total, 70% (268 patients) of the cohort exhibited type I ECG recordings, while 30% (115 patients) presented with a type II ECG pattern. Patients with type II ECGs were older (73.0 ± 11.1 vs. 67.8 ± 10.9; *p* < 0.001) and more frequently female (30.4% vs. 17.9%; *p* = 0.006). Regarding cardiovascular risk factors, diabetes mellitus was more prevalent in patients with type II ECGs (33.0% vs. 19.1%; *p* = 0.003). Furthermore, patients with type II ECGs had a higher CHA2DS2-VASc score (3.8 ± 1.7 vs. 2.7 ± 1.6; *p* < 0.001), a greater incidence of stroke history (15.7% vs. 6.7%; *p* = 0.006), and borderline significantly more often a diagnosis of AF compared to patients with type I ECGs (45.2% vs. 34.7%; *p* = 0.052). In 90.3% of cases, patients with type I ECGs exhibited CTI-dependent AFL, while patients with type II ECGs showed CTI-dependent atrial flutter in almost half of the cases (45.2%). Detailed information on patients’ characteristics compared between type I and type II ECGs is provided in Table 3.

#### 3.1.2. Baseline Characteristics of Patients with CTI-Dependent and Non-CTI-Dependent AFL

Among the cohort, 76.7% of the patients had CTI-dependent AFL, while 23.2% of the patients had non-CTI-dependent AFL. Patients with non-CTI-dependent AFL were older (74.1 ± 10.1 vs. 67.9 ± 11.1; *p* < 0.001) and more frequently female (40.4% vs. 16.0%, *p* < 0.001). Almost three-quarters (83.1%) of patients with non-CTI-dependent AFL were diagnosed with arterial hypertension, whereas patients with CTI-dependent AFL were significantly less likely to have arterial hypertension (68.4%). Patients with non-CTI-dependent AFL had a significantly higher CHA2DS2-VASc score compared to patients with CTI-dependent AFL (3.9 ± 1.7 vs. 2.8 ± 1.7; *p* < 0.001), were more frequently diagnosed with AF (50.6% vs. 34.0%; *p* = 0.005), had a higher prevalence of prior pulmonary vein isolation (20.2% vs. 5.8%, *p* < 0.001), were more frequently prescribed beta blockers (87.7% vs. 72.9%; *p* = 0.006), and had a higher incidence of stroke history (14.6% vs. 7.8%; *p* = 0.055). Among patients with CTI-dependent AFL, 82.3% showed a type I ECG, while 29.2% of patients with non-CTI-dependent AFL also exhibited a type I ECG. Detailed information on patient characteristics for those with CTI-dependent and non-CTI-dependent AFL is provided in Table 4.

#### 3.1.3. Baseline Characteristics of Subgroups Based on CTI Dependence and ECG Records

Based on the cross-tabulation (Table 1) mentioned earlier, four distinct subgroups can be identified: patients with CTI-dependent AFL and type I ECGs, patients with non-CTI-dependent AFL and type I ECGs, patients with CTI-dependent AFL and type II ECGs, and patients with non-CTI-dependent AFL and type II ECGs. Analysis of the patient characteristics of these groups revealed significant differences in age, sex, arterial hypertension, diabetes mellitus, beta-blocker intake, prior AF, prior pulmonary vein isolation, and prior heart surgery. Details are provided in Table 5.

### 3.2. Characteristics of the ECG and Electrophysiological Parameters Between Subgroups

The analysis of ECG and EPS characteristics between the subgroups during ongoing tachycardia demonstrated no statistically significant differences in terms of heart rate (*p* = 0.461) or QRS width (*p* = 0.315). The atrial CL in the group with CTI-dependent AFL and type II ECG was significantly longer (250.6 ± 44.1 ms) compared to the other groups (based on ECG characteristics: *p* = 0.023; EPS characteristics: *p* = 0.001). Furthermore, a statistically significant deviation was observed in the number of patients with an atrial CL over 245 ms (*p* ≤ 0.001) (Table 6).

### 3.3. Prediction Models for CTI-Dependent AFL and Type I ECG

Binary logistic regression revealed that female gender significantly decreases the likelihood of CTI-dependent AFL (*p* = 0.006). Furthermore, beta-blocker use (odds ratio 0.109, *p* = 0.014), a history of AF (odds ratio 0.415, *p* = 0.035), and previous pulmonary vein isolation (odds ratio 0.099, *p* < 0.001) significantly reduced the likelihood of CTI-dependent AFL (Table 7).

Multivariate analysis with the independent variable including patients with a history of AF, male gender increased the likelihood of CTI-dependent AFL by 201.6% (odds ratio 3.016, *p* = 0.019), and beta-blocker use reduced it by 87.8% (odds ratio 0.122, *p* = 0.043) (Table 8). Prior AF and prior PVI are interrelated and mutually influence each other, leading to potential multicollinearity. Therefore, only one variable was included to maintain statistical validity.

### 3.4. Prediction Models for CTI-Dependent AFL and Type II ECG

In the binary logistic regression analysis of patients with type II ECGs, arterial hypertension (odds ratio 0.375, *p* = 0.036), a higher CHA2DS2VASc score (odds ratio 0.758, *p* = 0.017), and increasing age were associated with a reduced likelihood of CTI-dependent AFL. In contrast, male gender (odds ratio 3.356, *p* = 0.007), prior cardiac surgery (odds ratio 3.056, *p* = 0.021), and longer atrial CL were associated with an increased likelihood of CTI-dependent AFL. Specifically, patients with an atrial CL > 245 ms were nearly three times more likely to develop CTI-dependent AFL and type II ECG compared to those with shorter atrial CL (Table 9).

**Figure 3:**
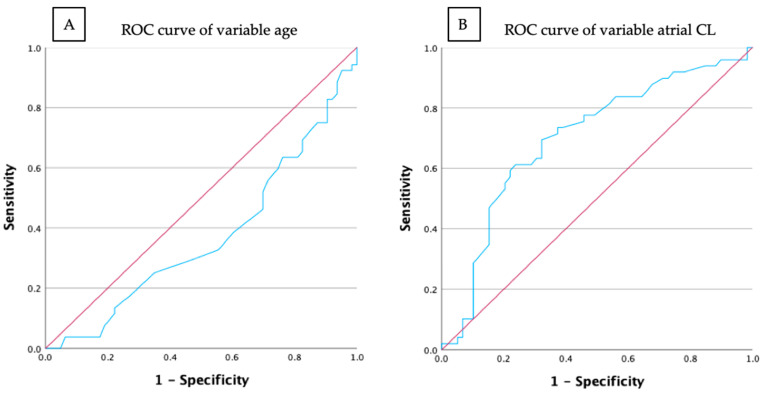
ROC curves for the predictive variables age and atrial cycle length. (**A**) ROC curve of the variable age (blue) and the reference line (red). (**B**) ROC curve of the cycle length measured during the EPS (blue) and the reference line (red). The x-axis represents 1-specificity (false positive rate), and the y-axis represents sensitivity (true positive rate). CL, cycle length; EPS, electrophysiological study; ROC, receiver operating characteristic.

The ROC analysis identified 75 years as the optimal threshold for differentiating CTI-dependent from CTI-independent AFL in patients with type II ECGs. However, the low AUC of 0.363 indicates limited discriminatory power of the parameter “Age > 75 years” for predicting CTI dependency. The ROC curve and the calculated AUC of 0.703 for the atrial CL measured during the EPS demonstrated superior discriminatory ability compared to age (Figure 3).

In the multivariate analysis, which included the significant variables from the binary logistic regression, male gender was associated with a 163.1% increase in the likelihood of CTI-dependent AFL (*p* = 0.045). Additionally, an atrial CL over 245 ms increased the likelihood of CTI-dependent AFL by 146.5% (*p* = 0.035). The remaining variables did not demonstrate statistical significance (Table 10).

## 4. Discussion

AFL is one of the most common cardiac arrhythmias, often occurring in combination with other arrhythmias such as AF [19,20]. Although it is characterized by a distinct electrophysiological substrate, it is frequently misdiagnosed by physicians [21,22]. Moreover, AFL is associated with elevated mortality rates, primarily due to its correlation with an increased risk of developing AF (50%), stroke, and other heart diseases [22,23,24,25]. Direct current cardioversion and pharmacological therapy are recommended for acute management of AFL, particularly in patients presenting with hemodynamic instability or symptomatic arrhythmia. While these modalities are effective for acute rhythm/rate control, catheter ablation is the most effective intervention for the long-term management of symptomatic and recurrent AFL episodes (class I recommendation) [16]. Consequently, in specialized centers, it is the most commonly performed procedure following pulmonary vein isolation [26,27,28]. However, there are various diagnostic modalities, making the differentiation between CTI-dependent and non-CTI-dependent AFL crucial for determining the optimal ablation approach. The aim of this study is to conduct a comprehensive analysis of the AFL cohort, encompassing patients with type I and type II ECGs as well as those with CTI-dependent and non-CTI-dependent AFL. Furthermore, the study aimed to identify clinical parameters with prognostic value for CTI dependence in AFL and to evaluate their predictive accuracy and diagnostic reliability.

In this study, patients with type I ECGs represented a younger and healthier cohort compared to those with type II ECGs, who had a higher prevalence of diabetes mellitus and stroke, as well as higher CHA_2_DS_2_-VASc scores. Similarly, CTI-dependent AFL was more prevalent in younger patients with fewer comorbidities. Compared to the study cohort of Barbato et al., where patients with prior valve interventions were excluded, non-CTI-dependent AFL in our study was significantly associated with older age [29]. The predictive relevance of age may result from an increased prevalence of cardiovascular diseases in older individuals, leading to atrial remodeling and favoring non-CTI-dependent conduction patterns [30]. Additional factors related to cardiac aging, such as oxidative stress, fibrosis, and slowed atrial conduction, may contribute to the formation of macro-reentrant tachycardias and atypical AFL pathways [31,32,33,34]. Although EPS remains the definitive method to confirm the mechanism of AFL, the clinical and electrophysiological predictors identified in our study are not intended as a substitute but rather as supportive tools to enhance diagnostic efficiency and patient stratification prior to invasive evaluation.

The current study demonstrated that additional clinical parameters also provide valuable predictive insights into the CTI dependence of AFL. In patients with type I ECGs, prior pulmonary vein isolation and AF significantly reduced the likelihood of CTI-dependent AFL, likely due to atrial remodeling induced by these conditions and their treatment [34,35,36]. Additionally, as previously reported by Gu et al., CTI-dependent AFL may present atypically on ECGs following ablation procedures in the left atrium, a finding that aligns with the results of this study [11]. Moreover, the use of beta blockers increased the probability of non-CTI-dependent AFL in patients with type I ECGs, possibly due to their negative bathmotropic effects on atrial conduction and their interaction with rate control in the context of AF management [37,38]. Male gender was strongly associated with CTI dependence in patients with both type I and type II ECGs, consistent with epidemiological data demonstrating a higher prevalence of AFL in men [1,16]. In contrast, women in this study tended to be older and more likely to present with non-CTI-dependent AFL, possibly due to secondary structural atrial remodeling linked to advanced age and cardiovascular comorbidities [30,34,39].

Longer atrial CL was identified as another significant predictive parameter for CTI dependence in patients with type II ECGs. Previous studies have shown a positive correlation between age and CL in CTI-dependent AFL, suggesting that age-related slowing of atrial conduction contributes to this association [32,40]. In the current study, ROC analysis of EPS-measured atrial CL demonstrated moderate discriminatory accuracy in differentiating CTI-dependent from CTI-independent AFL. However, the atrial CL was not a significant predictor of CTI dependence in patients with type I ECGs. Due to the retrospective nature of our study, only atrial CLs were systematically available for both CTI-dependent and non-CTI-dependent AFL. More detailed electrophysiological data, especially for the non-CTI-dependent group, were not uniformly recorded and thus could not be included.

The subsequent data analysis revealed that prior cardiac surgery increased the likelihood of CTI-dependent AFL in patients with type II ECGs, aligning with studies identifying CTI-dependent AFL as the most common atrial tachycardia following open-heart surgery. Postoperatively, larger atrial scars were associated with non-CTI-dependent AFL [41].

The final predictive factor analyzed in this study was arterial hypertension, which was associated with a reduced likelihood of CTI-dependent AFL in patients with type II ECGs. The increasing prevalence of hypertension with advancing age contributes to atrial remodeling processes, including the formation of low-voltage areas, thereby indicating a potential interaction between these two predictors [42].

The analysis presented in this study highlights the intricate interplay between clinical and electrophysiological parameters in predicting CTI dependence in AFL. A deeper understanding of these interactions has the potential to refine diagnostic criteria and guide the development of personalized therapeutic strategies. Key mechanistic factors, such as atrial scar burden or atrial size, were not systematically available in our retrospective cohort and should be incorporated in future studies. However, further investigation is required to validate these findings in larger cohorts, and improve the predictive accuracy for distinguishing CTI-dependent from non-CTI-dependent AFL.

### Limitations

As a retrospective, single-center analysis, this study may have limited generalizability. Although basic EPS parameters were included, detailed electrophysiological mapping data, especially for non-CTI-dependent AFL, were not uniformly available. Due to the observational design, causal relationships cannot be established.

## 5. Conclusions

In this study, the vast majority of patients with type I ECGs and approximately half of those with type II ECGs demonstrated CTI-dependent AFL. Nonetheless, well-defined clinical and electrophysiological predictors are crucial for improving diagnostic precision and optimizing therapeutic strategies. Our analysis identified male gender, absence of beta-blocker use, and no prior AF/PVI as the most reliable predictors of CTI dependence in patients with type I ECG AFL. For type II ECG AFL, significant predictors of CTI dependence included younger age, male gender, absence of arterial hypertension, lower CHA_2_DS_2_-VASc scores, prior cardiac surgery, and longer atrial CL. Coupling these parameters with ECG findings holds promise for refining prediction accuracy and optimizing patient care. Further studies are required to validate these findings, investigate additional predictive factors, and develop comprehensive scoring systems for more accurate risk stratification and individualized treatment approaches.

## Figures and Tables

**Table 1:** Cross-table of patients with CTI-dependent and non-CTI-dependent AFL categorized by type I and type II ECG findings.

	Type I ECG	Type II ECG	Total Participants
CTI-dependent AFL	242 (63.1)	52 (13.6)	294 (76.7)
Non-CTI-dependent AFL	26 (6.8)	63 (16.4)	89 (23.2)
Total participants	268 (70.0)	115 (30.0)	

A total of 268 patients showed type I ECGs and 115 patients showed type II ECGs, with AFL in 294 cases being CTI-dependent and 89 being non-CTI-dependent. AFL, atrial flutter; CTI, cavotricuspid isthmus; ECG, electrocardiogram.

**Table 2:** Baseline characteristics of the included patients with atrial flutter.

Baseline Characteristics	All Patients (*n* = 383)
Age [years], mean ± SD	69.3 ± 11.2
Male, *n* (%)	300 (78.3)
BMI [kg/m^2^], mean ± SD	28.1 ± 5.7
Coronary heart disease, *n* (%)	149 (39.2)
Arterial hypertension, *n* (%)	275 (71.8)
Hyperlipoproteinemia, *n* (%)	201 (52.5)
Diabetes mellitus, *n* (%)	89 (23.3)
Chronic obstructive pulmonary disease, *n* (%)	45 (11.7)
Obstructive sleep apnea syndrome, *n* (%)	22 (5.7)
Pulmonary hypertension, *n* (%)	27 (7.0)
Stroke, *n* (%)	36 (9.4)
Oral anticoagulation prior to ablation, *n* (%)	214 (58.0)
CHA_2_DS_2_-VASc score, mean ± SD	3.0 ± 1.7
Antiarrhythmic drugs - Antiarrhythmic drugs class III, *n* (%) - Beta blockers, *n* (%) - Digitalis glycosides, *n* (%)	31 (8.4) 281 (76.2) 24 (6.5)
Prior atrial fibrillation, *n* (%)	145 (37.9)
Prior PVI, *n* (%)	35 (9.1)
Prior LA/RA ablation (without PVI), *n* (%)	13 (3.4)
Prior heart surgery, *n* (%)	63 (16.4)

BMI, body mass index; LA, left atrial; PVI, pulmonary vein isolation; RA, right atrial; SD, standard deviation.

**Table 3:** Baseline characteristics of the patients with type I and type II ECGs.

	Type I ECG	Type II ECG	*p*-Value
Total, *n* (%)	268 (70.0)	115 (30.0)	N/A
Age [years], mean ± SD	67.8 ± 10.9	73.0 ± 11.1	<0.001
Male, *n* (%)	220 (82.1)	80 (69.6)	0.006
BMI [kg/m^2^], mean ± SD	28.1 ± 5.7	28.1 ± 5.8	0.920
Coronary heart disease, *n* (%)	97 (36.5)	52 (45.6)	0.094
Arterial hypertension, *n* (%)	185 (69.0)	90 (78.3)	0.066
Hyperlipoproteinemia, *n* (%)	136 (50.7)	65 (56.5)	0.300
Diabetes mellitus, *n* (%)	51 (19.1)	38 (33.0)	0.003
Chronic obstructive pulmonary disease, *n* (%)	33 (12.3)	12 (10.4)	0.601
Obstructive sleep apnea syndrome, *n* (%)	15 (5.6)	7 (6.1)	0.850
Pulmonary hypertension, *n* (%)	17 (6.3)	10 (8.7)	0.410
Stroke, *n* (%)	18 (6.7)	18 (15.7)	0.006
Oral anticoagulation prior to ablation, *n* (%)	148 (56.5)	66 (61.7)	0.359
CHA_2_DS_2_-VASc score, mean ± SD	2.7 ± 1.6	3.8 ± 1.7	<0.001
Antiarrhythmic drugs - Antiarrhythmic drugs class III, *n* (%) - Beta blockers, *n* (%) - Digitalis glycosides, *n* (%)	25 (9.5) 193 (73.7) 17 (6.5)	6 (5.6) 88 (82.2) 7 (6.5)	0.216 0.079 0.985
Prior atrial fibrillation, *n* (%)	93 (34.7)	52 (45.2)	0.052
Prior PVI, *n* (%)	21 (7.8)	14 (12.2)	0.177
Prior heart surgery, *n* (%)	39 (14.6)	24 (20.9)	0.126
CTI-dependent atrial flutter, *n* (%)	242 (90.3)	52 (45.2)	<0.001

BMI, body mass index; CTI, cavotricuspid isthmus; ECG, electrocardiogram; N/A, not applicable; PVI, pulmonary vein isolation; SD, standard deviation.

**Table 4:** Baseline characteristics of the patients with CTI-dependent and non-CTI-dependent AFL.

	CTI	Non-CTI	*p*-Value
Total, *n* (%)	294 (76.7)	89 (23.2)	*n*/A
Age [years], mean ± SD	67.9 ± 11.1	74.1 ± 10.1	<0.001
Male, *n* (%)	247 (84.0)	53 (59.6)	<0.001
BMI [kg/m^2^], mean ± SD	28.0 ± 5.3	28.5 ± 7.1	0.544
Coronary heart disease, *n* (%)	107 (36.6)	42 (47.7)	0.478
Arterial hypertension, *n* (%)	201 (68.4)	74 (83.1)	0.007
Hyperlipoproteinemia, *n* (%)	147 (50.0)	54 (60.7)	0.077
Diabetes mellitus, *n* (%)	65 (22.2)	24 (27.0)	0.350
Chronic obstructive pulmonary disease, *n* (%)	38 (12.9)	7 (7.9)	0.194
Obstructive sleep apnea syndrome, *n* (%)	18 (6.1)	4 (4.5)	0.563
Pulmonary hypertension, *n* (%)	21 (7.1)	6 (6.7)	0.897
Stroke, *n* (%)	23 (7.8)	13 (14.6)	0.055
Oral anticoagulation prior to ablation, *n* (%)	161 (55.9)	53 (65.4)	0.125
CHA_2_DS_2_-VASc score, mean ± SD	2.8 ± 1.7	3.9 ± 1.7	<0.001
Antiarrhythmic drugs - Antiarrhythmic drugs class III, *n* (%) - Beta blockers, *n* (%) - Digitalis glycosides, *n* (%)	25 (8.7) 210 (72.9) 18 (6.3)	6 (7.4) 71 (87.7) 6 (7.4)	0.751 0.006 0.709
Prior atrial fibrillation, *n* (%)	100 (34.0)	45 (50.6)	0.005
Prior PVI, *n* (%)	17 (5.8)	18 (20.2)	<0.001
Prior heart surgery, *n* (%)	49 (16.7)	14 (15.7)	0.835
ECG, type I, *n* (%)	242 (82.3)	26 (29.2)	<0.001

AFL, atrial flutter; BMI, body mass index; CTI, cavotricuspid isthmus; ECG, electrocardiogram; N/A, not applicable; PVI, pulmonary vein isolation; SD, standard deviation.

**Table 5:** Baseline characteristics of the subgroups based on CTI dependence and ECG records.

	CTI + Type I ECG	Non-CTI + Type I ECG	CTI + Type II ECG	Non-CTI + Type II ECG	*p*-Value
Total, *n* (%)	242 (63.1)	26 (6.8)	52 (13.6)	63 (16.4)	N/A
Age [years], mean ± SD	67.4 ± 10.7 *^,^***	71.1 ± 11.9 *	70.1 ± 12.6 ^#^	75.4 ± 9.0 ***^,#^	0.001
Male, *n* (%)	204 (84.3) **^,^***	16 (61.5) **	43 (82.7) *	37 (58.7) ***^,^*	0.001
BMI [kg/m^2^], mean ± SD	28.0 ± 5.4	29.6 ± 7.9	28.2 ± 4.4	28.0 ± 6.8	0.600
Coronary heart disease, *n* (%)	85 (35.1)	14 (53.8)	24 (46.2)	28 (41.9)	0.117
Arterial hypertension, *n* (%)	165 (68.2) *	20 (76.9)	36 (69.2)	54 (85.7) *	0.044
Hyperlipoproteinemia, *n* (%)	119 (49.2)	17 (65.4)	28 (53.8)	37 (58.7)	0.259
Diabetes mellitus, *n* (%)	49 (20.2) *^,^**	2 (7.7) ^#^	16 (30.8) *	22 (34.9) **^,#^	0.013
COPD, *n* (%)	31 (12.8)	2 (7.7)	7 (13.5)	5 (7.9)	0.638
OSAS, *n* (%)	13 (5.4)	2 (7.7)	5 (9.6	2 (3.2)	0.505
Pulmonary hypertension, *n* (%)	15 (6.2)	2 (7.7)	6 (11.5)	4 (6.3)	0.617
Stroke, *n* (%)	16 (6.7) *	2 (7.7)	7 (13.5)	11 (17.5) *	0.047
OAC prior ablation, *n* (%)	132 (55.5)	16 (61.5)	29 (55.8)	37 (64.9)	0.458
CHA_2_DS_2_-VASc score, mean ± SD	2.6 ± 1.6 *^,^***^,###^	3.3 ± 1.5 *	3.4 ± 1.8 ***^,#^	4.2 ± 1.6 ^#,###^	0.001
Antiarrhythmic drugs - AA class III, *n* (%) - Beta blockers, *n* (%) - Digitalis glycosides, *n* (%)	22 (9.2) 170 (70.2) *^,#^ 16 (6.6)	3 (12.5) 23 (95.8) * 1 (4.2)	3 (5.8) 40 (76.9) 2 (3.8)	3 (5.3) 48 (84.2) ^#^ 5 (8.8)	0.591 0.013 0.734
Prior atrial fibrillation, *n* (%)	79 (32.6) *^,#^	14 (53.8) *	21 (40.4)	31 (49.2) ^#^	0.029
Prior PVI, *n* (%)	12 (5.0) *^,^***	9 (34.6) ***	5 (9.6)	9 (14.3) *	0.001
Prior heart surgery, *n* (%)	33 (13.6) ***	6 (23.1)	16 (30.8) ***	8 (12.7)	0.005

*p*-values on the right side of the table indicate overall comparisons between the four defined subgroups using ANOVA or chi-square tests, as appropriate. For pairwise between-group comparisons, additional symbols are used to indicate levels of statistical significance: * and ^#^ for *p* < 0.05, ** for *p* < 0.01, and *** and ^###^ for *p* < 0.001. AAs, antiarrhythmics; COPD, chronic obstructive pulmonary disease; ECG, electrocardiogram; N/A, not applicable; OAC, oral anticoagulation; OSAS, obstructive sleep apnea syndrome; PVI, pulmonary vein isolation; SD, standard deviation.

**Table 6:** Comparison of electrophysiological and ECG characteristics between the subgroups.

	Total	CTI + Type I ECG	Non-CTI + Type I ECG	CTI + Type II ECG	Non-CTI + Type II ECG	*p*-Value
ECG characteristics						
Heart rate [bpm], mean ± SD	98.2 ± 31.3	96.6 ± 32.5	98.7 ± 33.3	98.8 ± 32.7	103.6 ± 29.4	0.461
QRS [ms], mean ± SD	113.0 ± 32.2	112.4 ± 32.5	110.5 ± 34.8	120.4 ± 32.4	110.4 ± 29.2	0.315
Atrial CL [ms], mean ± SD	234.3 ± 39.8	232.6 ± 36.0	224.6 ± 31.3 *^,#^	250.6 ± 44.1 *	231.1 ± 49.9 ^#^	0.023
Atrial CL > 245 ms, *n* (%)	121 (31.7)	69 (28.5) ***	6 (23.1) **	28 (53.8) ***^,^**	18 (29.0)	<0.001
Ventricular PM stimulation, *n* (%)	23 (6.0)	13 (5.4)	2 (7.7)	6 (11.5)	2 (3.2)	0.286
EPS characteristics						
Atrial CL [ms], mean ± SD	244.8 ± 33.7	241.6 ± 32.2 **	238.0 ± 22.4 *	264.7 ± 36.4 **^,^*	243.1 ± 35.6	0.001
Atrial CL > 245 ms, *n* (%)	163 (45.2)	97 (40.1) **^,^*	9 (39.1)	35 (71.4) **^,^*	22 (37.3)	0.001

*p*-values on the right side of the table indicate overall comparisons between the four defined subgroups using ANOVA or chi-square tests, as appropriate. For pairwise between-group comparisons, additional symbols are used to indicate levels of statistical significance: * and ^#^ for *p* < 0.05, ** for *p* < 0.01, and *** for *p* < 0.001. Bpm, beats per minute; CL, cycle length; CTI, cavotricuspid isthmus; ECG, electrocardiogram; EPS, electrophysiological study; PM, pacemaker; SD, standard deviation.

**Table 7:** Binary logistic regression analysis of the type I ECG group to evaluate predictors of CTI-dependent AFL.

	Odds Ratio	95% Confidence Interval	*p*-Value
Age [years]	0.965	0.924–1.007	0.100
Age > 75 years	0.600	0.259–1.389	0.233
Male	3.356	1.416–7.937	0.006
Body mass index [kg/m^2^]	0.960	0.906–1.019	0.180
Arterial hypertension	0.643	0.248–1.665	0.363
Hyperlipoproteinemia	0.512	0.220–1.194	0.121
Diabetes mellitus	3.062	0.700–13.402	0.137
Coronary heart disease	0.464	0.205–1.048	0.065
CHA_2_DS_2_VASc score [points]	0.793	0.621–1.013	0.063
AAT with beta blockers	0.109	0.014–0.821	0.031
Prior atrial fibrillation	0.415	0.184–0.940	0.035
Prior PVI	0.099	0.036–0.266	<0.001
Prior heart surgery	0.526	0.197–1.407	0.201
Atrial CL [ms]	1.004	0.990–1.018	0.591
Atrial CL > 245 ms	1.135	0.472–2.728	0.778

AAT, antiarrhythmic therapy; PVI, pulmonary vein isolation.

**Table 8:** Coefficient table for multivariate analysis of CTI-dependent atrial flutter and type I ECG with the independent variable “Prior atrial fibrillation”.

	Regression Coefficient	Standard Error	Wald	df	Sig.	Odds Ratio	95% Confidence Interval
Male	1.104	0.470	5.505	1	0.019	3.016	1.199–7.583
AAT with beta blockers	−2.102	1.037	4.109	1	0.043	0.122	0.016–0.933
Prior PVI	−0.754	0.444	2.880	1	0.090	0.470	0.197–1.124

AAT, antiarrhythmic therapy; df, degrees of freedom; PVI, pulmonary vein isolation; Sig., significance.

**Table 9:** Binary logistic regression analysis of the type II ECG group to evaluate predictors of CTI-dependent AFL.

	Odds Ratio	95% Confidence Interval	*p*-Value
Age [years]	0.954	0.918–0.991	0.016
Age > 75 years	0.370	0.172–0.796	0.011
Male	3.356	1.399–8.065	0.007
Body mass index [kg/m^2^]	1.003	0.937–1.074	0.926
Arterial hypertension	0.375	0.150–0.940	0.036
Hyperlipoproteinemia	0.820	0.391–1.720	0.599
Diabetes mellitus	0.828	0.378–1.815	0.638
Coronary heart disease	1.005	0.481–2.099	0.990
CHA_2_DS_2_VASc score [points]	0.758	0.604–0.951	0.017
AAT with beta blockers	0.433	0.080–2.339	0.331
Prior atrial fibrillation	0.699	0.333–1.469	0.345
Prior PVI	0.638	0.200–2.038	0.449
Prior heart surgery	3.056	1.185–7.877	0.021
Atrial CL [ms]	1.017	1.005–1.029	0.004
Atrial CL > 245 ms	4.205	1.863–9.489	<0.001

AAT, antiarrhythmic therapy; CL, cycle length; PVI, pulmonary vein isolation.

**Table 10:** Coefficient table for multivariate analysis of CTI-dependent atrial flutter and type II ECG.

	Regression Coefficient	Standard Error	Wald	df	Sig.	Odds Ratio	95% Confidence Interval
Age [years]	−0.032	0.022	2.073	1	0.150	0.969	0.927–1.012
Male	0.967	0.482	4.030	1	0.045	2.631	1.023–6.765
Arterial hypertension	−0.524	0.536	0.955	1	0.328	0.592	0.207–1.693
Prior heart surgery	0.547	0.531	1.062	1	0.303	1.728	0.610–4.895
Atrial CL > 245 ms	0.902	0.428	4.451	1	0.035	2.465	1.066–5.701

CL, cycle length; df, degrees of freedom; Sig., significance.

## Data Availability

The data presented in this study are available on request from the corresponding author. The data are not publicly available due to data privacy laws.

## Abbreviations

The following abbreviations are used in this manuscript:

AAs	Antiarrhythmics
AAT	Antiarrhythmic therapy
AF	Atrial fibrillation
AFL	Atrial flutter
ANOVA	Analysis of variance
AUC	Area under the curve
BMI	Body mass index
Bpm	Beats per minute
CCW	Counterclockwise
CL	Cycle length
COPD	Chronic obstructive pulmonary disease
CS	Coronary sinus
CTI	Cavotricuspid isthmus
CW	Clockwise
Df	Degrees of freedom
ECG	Electrocardiogram
EPS	Electrophysiological study
ICD	International classification of diseases
IVC	Inferior vena cava
LA	Left atrial
LAO	Left anterior oblique
LRA	Low right atrium
N/A	Not applicable
OAC	Oral anticoagulation
OR	Odds ratio
OSAS	Obstructive sleep apnea syndrome
PM	Pacemaker
PPI	Post-pacing interval
PVI	Pulmonary vein isolation
RA	Right atrial
ROC	Receiver operator characteristics
SD	Standard deviation
Sig.	Significance
